# Transcriptome analysis reveals defense responses of alfalfa seedling roots to *Sclerotium rolfsii*

**DOI:** 10.3389/fpls.2025.1561723

**Published:** 2025-04-15

**Authors:** Shizhen Jia, Zhencuo Dan, He Li, Yuhan Guo, Lei Jia, Ailing Yu, Huitong Zhan, Xiangjun Liu, Teng Gao, Yun Shi, Zeng-Yu Wang, Lili Cong

**Affiliations:** ^1^ College of Grassland Science, Qingdao Agricultural University, Qingdao, China; ^2^ Key Laboratory of National Forestry and Grassland Administration on Grassland Resources and Ecology in Yellow River Delta, Qingdao Agricultural University, Qingdao, China; ^3^ Hinggan League Institute of Agricultural and Husbandry Sciences, Ulanhot, China; ^4^ Inner Mongolia Innovation Center of Biological Breeding Technology, Ulanhot, China

**Keywords:** alfalfa, Sclerotium rolfsii, transcriptome sequencing, WGCNA, response mechanism

## Abstract

**Introduction:**

*Sclerotium rolfsii* is a major pathogen responsible for root rot in various plants, including alfalfa (*Medicago sativa*). Additionally, this pathogen can also cause root diseases in alfalfa relatives, such as *Medicago truncatula*, soybean (*Glycine max*), and mung bean (*Vigna radiata*). This study aims to explore the interaction mechanisms between alfalfa and S. rolfsii, identify key regulatory factors involved in disease resistance, and provide insights for improving alfalfa's resistance to root rot.

**Methods:**

In this study, the *S. rolfsii* strain CZL1 was isolated and identified as the primary pathogen responsible for root rot outbreaks in Qingdao, Shandong Province. *M. sativa* cv. WuDi was used as the experimental material. After inoculating the plants with strain CZL1, root samples were collected at 24 hours post-inoculation (hpi) and 4 days post-inoculation (dpi) for transcriptome sequencing.

**Results:**

A total of 11,433 and 12,063 differentially expressed genes (DEGs) were identified at CK (Control, non-inoculated) versus T24 h (24 hpi) and CK versus T4 d (4 dpi), respectively. Plant hormone signal transduction pathways exhibited the highest number of DEGs at 24 hpi, while plant-pathogen interaction pathways were dominant at 4 dpi. Key genes in these pathways include PR-1 (Pathogenesis-Related protein 1), PPR (Pentatricopeptide Repeat protein), and F-box (F-box Kelch-repeat protein). Additionally, the phenylpropanoid biosynthesis pathway, which is involved in lignin and flavonoid synthesis, plays a crucial role in disease resistance. Important genes involved in this pathway, such as PAL, C4H, 4CL, CHS, and CHI, were found to be significantly enriched. Furthermore, the WRKY transcription factor family was identified as a key regulator of multiple metabolic pathways related to disease resistance.

**Conclusion:**

The findings provide a comprehensive understanding of the key molecular factors involved in alfalfa’s response to *S. rolfsii* infection, laying a theoretical foundation for future research on disease resistance mechanisms in alfalfa.

## 1 Introduction

Alfalfa (*Medicago sativa* L.) is a leguminous forage crop renowned for its high yield, excellent palatability, and wide adaptability. Additionally, alfalfa has a strong root system and nitrogen-fixing ability, making it a valuable green manure crop (Wang et al., 2023). The increasing alfalfa planting areas and the uniformity of planting methods have caused a steady rise in disease incidence (Yang et al., 2022a). New disease types are gradually emerging. For instance, root diseases caused by *S. rolfsii* (Pratt and Rowe, 2002; Liu et al., 2021), *Fusarium* spp (Abbas et al., 2022; Li et al., 2022), *Phytophthora* spp (Midgley et al., 2022), *Trichothecium roseum* (Liu et al., 2024) has significantly limited the yield and quality of alfalfa. However, southern blight caused by *S. rolfsii* is the major root disease in temperate and tropical alfalfa-growing regions, exacerbated by climate change and mechanized farming practices. In 2019, 70-90% of regreened alfalfa plants in the alfalfa-growing region of Jiaozhou (Qingdao, Shandong Province) suffered root rot, withered, and died. Morphological and molecular biological identification confirmed that the disease was Southern wilt, caused by *Sclerotium rolfsii* (Liu et al., 2021). From 2020 to 2022, disease outbreaks continued in the same region.

*S. rolfsii* is a widespread soil-borne fungal pathogen with strong saprophytic abilities and a broad host range, infecting over 500 plant species across nearly 100 families, including dicotyledonous and monocotyledonous plants. Thus, it significantly damages major cash crops such as wheat (*Triticum aestivum*) (Choppakatla et al., 2006), peanut (*Arachis hypogaea*) (Jogi et al., 2016; Han et al., 2023), and soybean (*Glycine max*) (Zheng et al., 2021). New hosts are still being reported. Additionally, *S. rolfsii* has a vast distribution range, predominantly in the Americas, Europe, Asia, and several tropical and subtropical regions characterized by high temperatures and high humidity (Polizzi et al., 2010). The pathogen primarily infects plant roots and stems but may invade fruits, leaves, petioles, flowers, and other plant parts, causing diseases such as root rot, stem rot, leaf spot, leaf blight, and southern blight.

Since 1966, alfalfa southern blight disease caused by *S. rolfsii* has been found in Jilin province, China, and spread to Shandong province (China) (Liu et al., 2021). Alfalfa diseases primarily occur from the branching to the budding stages. Similar to cowpea (*Vigna unguiculata*) (Huang et al., 2023), the initial symptoms of the disease manifest as brown, water-soaked spots at the base of the stem or root collar, which gradually expand into the internal tissues, leading to the formation of brown necrotic lesions on the roots. During this stage, the above-ground parts of the plant wilt during the day but recover at night and in the early morning. As the disease progresses, the stem base begins to split, exposing the internal vascular tissues. In the later stages, a white mycelial layer develops on the surface of the infected area, with hyphae spreading into the surrounding soil, turning it white. As the diseased plants die, the hyphae turn brown and produce numerous brown sclerotia on the affected surface. These brown sclerotia persist in the soil or on plant debris over the winter and can reinfect new plants in the following growing season.

Despite recent research on Southern wilt disease in alfalfa focusing primarily on pathogen isolation, identification, and control, the underlying mechanisms governing the *S. rolfsii*-alfalfa interaction remain unclear. To address this gap, this study examined the roots of *M. sativa* cv. WuDi at two time points post-infection with *S. rolfsii* and conducted transcriptome sequencing to explore the resistance mechanisms involved in the *S. rolfsii*-alfalfa interaction. The findings enhance our understanding of the complexity of plant-pathogen interactions and provide a solid scientific foundation for developing alfalfa cultivars with improved resistance to *S. rolfsii* in the future.

## 2 Materials and methods

### 2.1 Pathogenicity analysis of *Sclerotium rolfsii* CZL1

Pathogenicity analysis was conducted following the Zhang method (Zhang et al., 2022) using *S. rolfsii* strain CZL1. Two hundred uniform-sized oat (*Avena sativa*) seeds were placed in conical flasks and soaked in water overnight to remove excess water. After autoclaving twice, four 1cm-wide agar blocks inoculated with the pathogen were added to each flask. When the oat seeds were fully inoculated (approximately 3 weeks), 20-day-old potted alfalfa, soybean, *M. truncatula*, and mung bean (*Vigna radiata*) were inoculated by placing two fungi-inoculated oat seeds 1 cm below the root crown. The controls were inoculated with sterilized oat seeds that were not fungi-infected. After 20 days, pathogenicity was recorded, and the incidence rate and disease severity index were calculated. All experiments were performed in triplicate.


$$\begin{array}{c} Disease\;incidence\left(DI\right)\\ =\;\left(number\;of\;infected\;plants/total\;number\;of\;infected\;plants\right)\;\\ \times \;100\% \end{array}$$

$$\begin{array}{c} Disease\;severity\;index\left(DSI\right)\\ =\sum (number\;of\;infected\;plants\;at\;all\;levels\\ \;\times \;value\;of\;disease\;grade)/(total\;number\;of\;investigated\;plants\\ \;\times \;highest\;value)\;\times \;100\% \end{array}$$

### 2.2 Plant materials, preparation, inoculation, and sample collection

The plant material used in this study was M. sativa cv. WuDi. The control and inoculated samples were maintained in artificial climate incubator set to 27°C (during the day) and 25°C (at night), with a 16-hour light cycle and 95% relative humidity. For the control group, sterilized oat seeds were buried 2 cm deep in the soil around the base of the root crown. In the treatment group, oat seeds inoculated with *S. rolfsii* were buried at the same depth, with two seeds per plant. Roots samples were collected at 24 hours post-inoculation (hpi) and 4 days post-inoculation (dpi) and rinsed three times with sterile water. Next, the root was cut into ~ 1 mm segments, placed in sterile 2 mL centrifuge tubes, snap-frozen in liquid nitrogen, and stored at - 80°C. The samples were subsequently analyzed via transcriptome sequencing and validated using qRT-PCR.

### 2.3 RNA extraction, cDNA library construction, and Illumina sequencing

Total RNA was extracted using the TaKaRa MiniBEST Plant RNA Extraction Kit (TAKARA Material Technology Co., Ltd., Province/State/City, Country). The quality of the extracted RNA was assessed using 1% agarose gel electrophoresis, while, RNA concentration and purity were measured using NanoDrop 2000 (Thermo Fisher Scientific, MA, USA). RNA integrity was assessed using the RNA Nano 6000 Assay Kit of the Agilent Bioanalyzer 2100 system (Agilent Technologies, CA, USA). Next, 1 μg RNA per sample was used for the RNA sample preparations. Sequencing libraries were generated following the manufacturer’s protocol using the NEBNext UltraTM RNA Library Prep Kit for Illumina (NEB, MA, USA). Index codes were added to assign sequences to each sample. A total of nine samples were collected, including RNA samples from M. sativa cv. WuDi at 24 hours post-inoculation (hpi) and 4 days post-inoculation (dpi) with *S. rolfsii*, as well as control samples (each group containing three biological replicates), were sequenced using the Illumina platform at BMK Cloud (www.biocloud.net) Biotechnology Co., Ltd.

### 2.4 Bioinformatics analysis

Raw fastq reads were initially processed using in-house Perl scripts to remove adapter sequences and filter out reads containing poly-N and low-quality bases. Quality metrics, including Q20, Q30, GC content, and sequence duplication levels were calculated to ensure that downstream analyses were conducted on clean, high-quality data.

The filtered high-quality, reads were then mapped to the *M. sativa* cv. XinJiangDaYe reference genome. Only perfectly matched reads or those with a single mismatch were further analyzed and annotated based on the reference genome.

Differentially expressed genes (DEGs) were identified based on fold change (FC) and *p*-value difference. The screening criteria were set as a fold change (≥2) and false discovery rate (FDR<0.05). The filtered DEGs were subjected to the Gene Ontology database (GO, http://www.geneontology.org/), COG (http://www.ncbi.nlm.nih.gov/COG/), and Kyoto Encyclopedia of Genes and Genomes (KEGG, http://www.genome.jp/kegg/) analyses. Functional enrichment analysis was performed to identify the biological functions and metabolic pathways influenced by the differentially expressed genes. All relevant project information, including sample details, raw data, quality control metrics, and comparative analysis results, can be found in **Supplementary Table 1**.

The highly co-expressed gene modules were inferred from the DEGs using weighted gene co-expression network analysis (WGCNA), an R package (Langfelder and Horvath, 2008). WGCNA network construction and module detection were conducted using an unsigned topological overlap matrix (TOM), a power β of 5 and a branch merge cut height of 0.25. The module eigengene (the first principal component of a given module) value was calculated and used to evaluate the association between the modules and anthocyanin content across the 9 samples.

### 2.5 Alignment of reference sequences

The sequences were mapped to the *M. sativa* cv. XinJiangDaYe reference genome (https://figshare.com/articles/dataset/genome_fastasequenceandnnotationfiles/12327602). The HISAT2 (version 2.0.4) (Kim et al., 2015) software was used to obtain annotation information about new genes and feature information characterized by sequence samples. Sequences aligned to the reference genome were classified as mapped reads, which served as the basis for subsequent analyses. The suitability of the samples was assessed based on the genome mapping rate.

### 2.6 Real-time quantitative PCR analysis of the DEGs

qRT-PCR experiments were conducted using the RNA samples returned by the sequencing company after transcriptome sequencing. The reactions were performed with the TB Green Premix Ex TaqTM II kit (Tli RNaseHPlus) (TaKaRa, Kusatsu, Tokyo, Japan) on a CFX-96 device. Next, a 1μg sample of total RNA was used for cDNA synthesis using the PrimeScriptTMRT reagent kit with gDNA Eraser (TaKaRa, Kusatsu, Tokyo, Japan). An Ultra-micro spectrophotometer was used to quantitate 100 ng of single-stranded cDNA which was subsequently used for qRT-PCR analysis. Relative quantitative analysis was performed under the following conditions: 95°C for 30 s and 40 cycles at 95°C 5 s, 60°C 30 s.

A melting curve analysis, from 65°C to 95°C, was used to identify different amplicons, including non-specific products. Three technical replicates were employed for each tested sample and template-free negative controls. Gene-specific primers were designed by Primer 5 software (**Supplementary Table 2**). Relative quantification was normalized using the *β-actin* housekeeping control gene, and the fold change in gene expression was calculated using the 2^-ΔΔt^ method.

## 3 Result and analysis

### 3.1 Pathogenicity analysis of *Sclerotium rolfsii* CZL1

The disease incidence on alfalfa, soybean, *M. truncatula*, and mung bean was observed after inoculation (1cm below the root crown). The disease incidence, disease severity (grades 0, 1, 2, and 3), disease severity index, plant height, and root length were assessed 20 days after inoculation.

**Figure 1:**
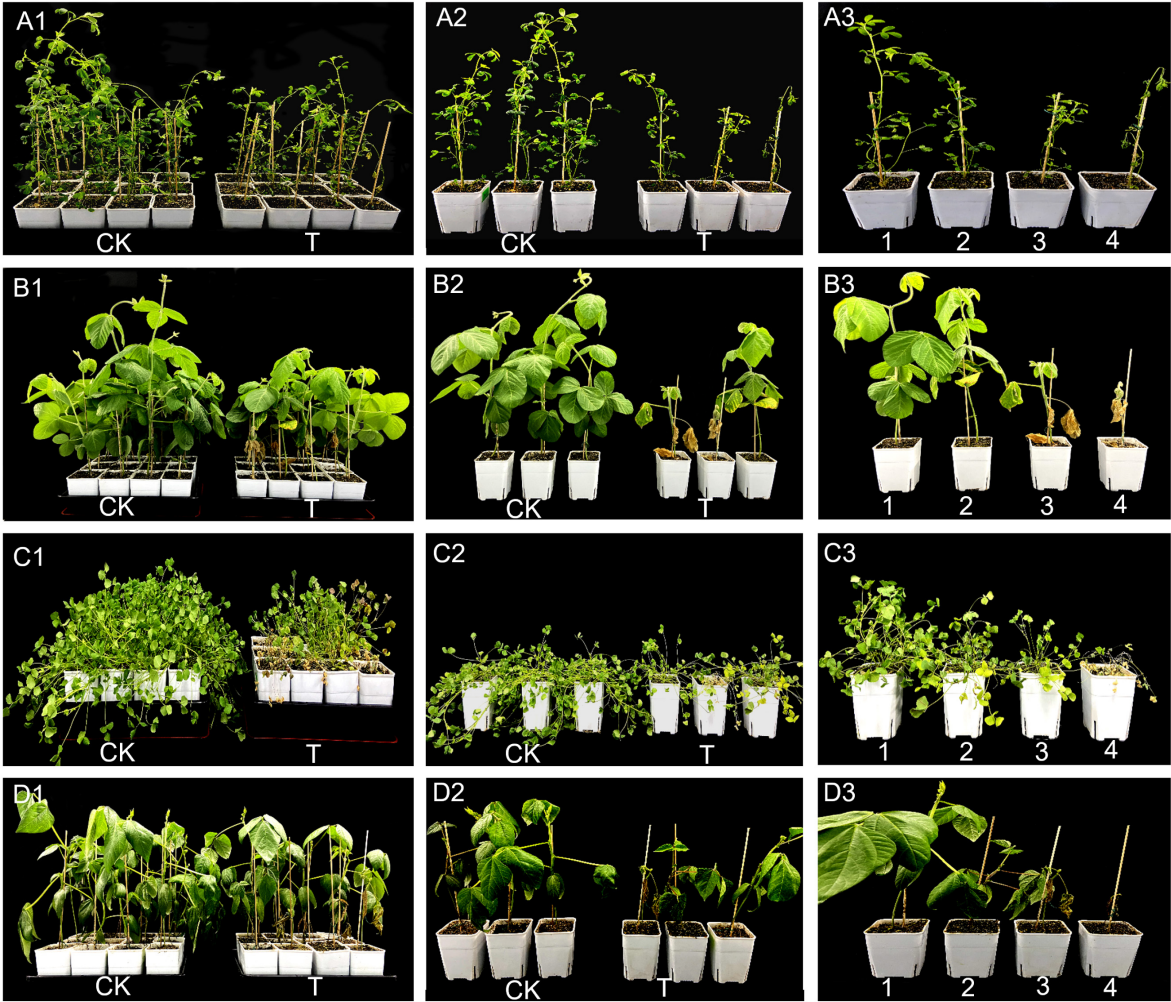
Pathogenicity of *S. rolfsii* in four legumes. **(A-D)** Represent alfalfa, soybean, M. truncatula, and mung bean, respectively. **(A1-D1)** and **(A2-D2)** Phenotypic comparison of the four plant species under untreated conditions (CK) and pathogen-inoculated conditions (T) at 20 days post-inoculation. **(A3-D3)** Control chart illustrating the disease severity levels in the four plant species following pathogen infection.

**Figure 2:**
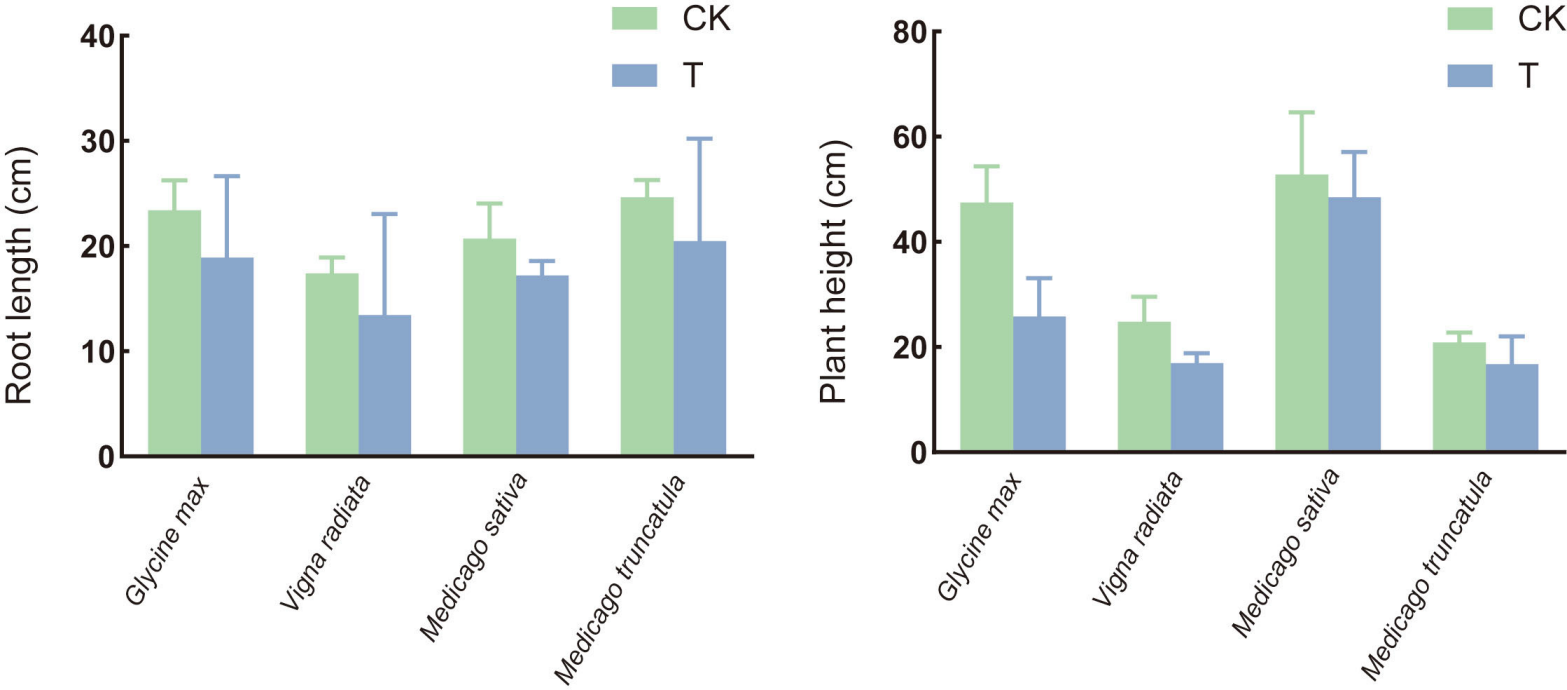
Effects of *S. rolfsii* on the plant height and root length of soybean (*Glycine max*), mung bean (*Vigna radiata*), alfalfa (*Medicago sativa*), *and M. truncatula.* CK: uninoculated plant. T: inoculated plant.

The pathogenicity varied among the four legume species, with alfalfa and mung bean exhibiting a disease severity index of over 60% and an incidence rate exceeding 87% (**Figure 1**). Infection significantly reduced plant height and root length in all four plant species, showing a marked growth inhibition effect compared to the control group (**Figure 2**).

### 3.2 Phenotypic characteristics of alfalfa after inoculation

**Figure 3:**
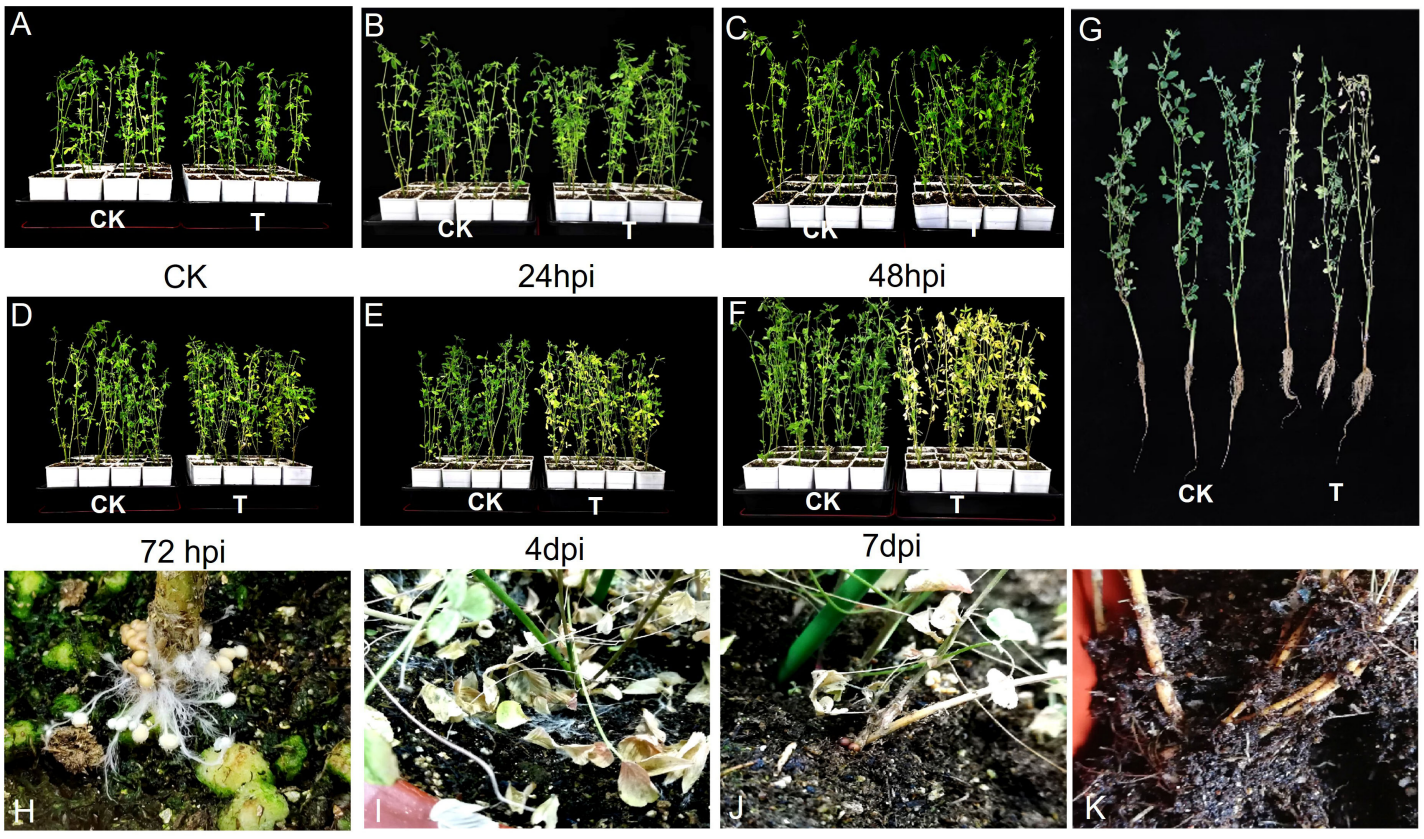
Morphological observation of *M. sativa* cv. WuDi inoculated with *S. rolfsii* ***(*A*)*** Alfalfa seedlings before inoculation. **(B-F)** Morphology of alfalfa plants at 24 hpi, 48 hpi, 72 hpi, 4 dpi, and 7 dpi. **(G)** Comparison between healthy and inoculated alfalfa leaves. **(H, J, K)** Sclerotium at different stages. **(I)** Mycelium. RNA-Seq and results.

The inoculated gradually withered and died as the inoculation time extended (**Figure 3**). At 24 hpi, there was no significant difference in the phenotypes of the control and inoculated groups. After 48 hpi, the inoculated plants began to show signs of wilting, with the roots exhibiting slight discoloration in the early stage (pale yellow patches), followed by clear wilting and yellowing. At the late stage, 4 days post-infection, the plant roots showed dark brown patches attached to the white mycelium layer. The white mycelium spreads around the alfalfa stem (**Figure 3H**) and on the surface of the nutritive soil. Hyphal growth was also observed at the base of the stem (**Figure 3I-J**). In contrast, the control group grew well. Infection points can appear 24 h after inoculation, with typical disease spots visible 4-6 days later (Zhang et al., 2022). Based on these observations, roots sampled at 24 h and 4 d after inoculation were selected for transcriptome sequencing (**Figure 3**).

### 3.3 RNA-Seq and results 

Plant RNA was extracted using the Trizol method, and its integrity was verified by gel electrophoresis. The RNA displays clear, bright bands, meeting the requirements for library construction and sequencing.

**Figure 4:**
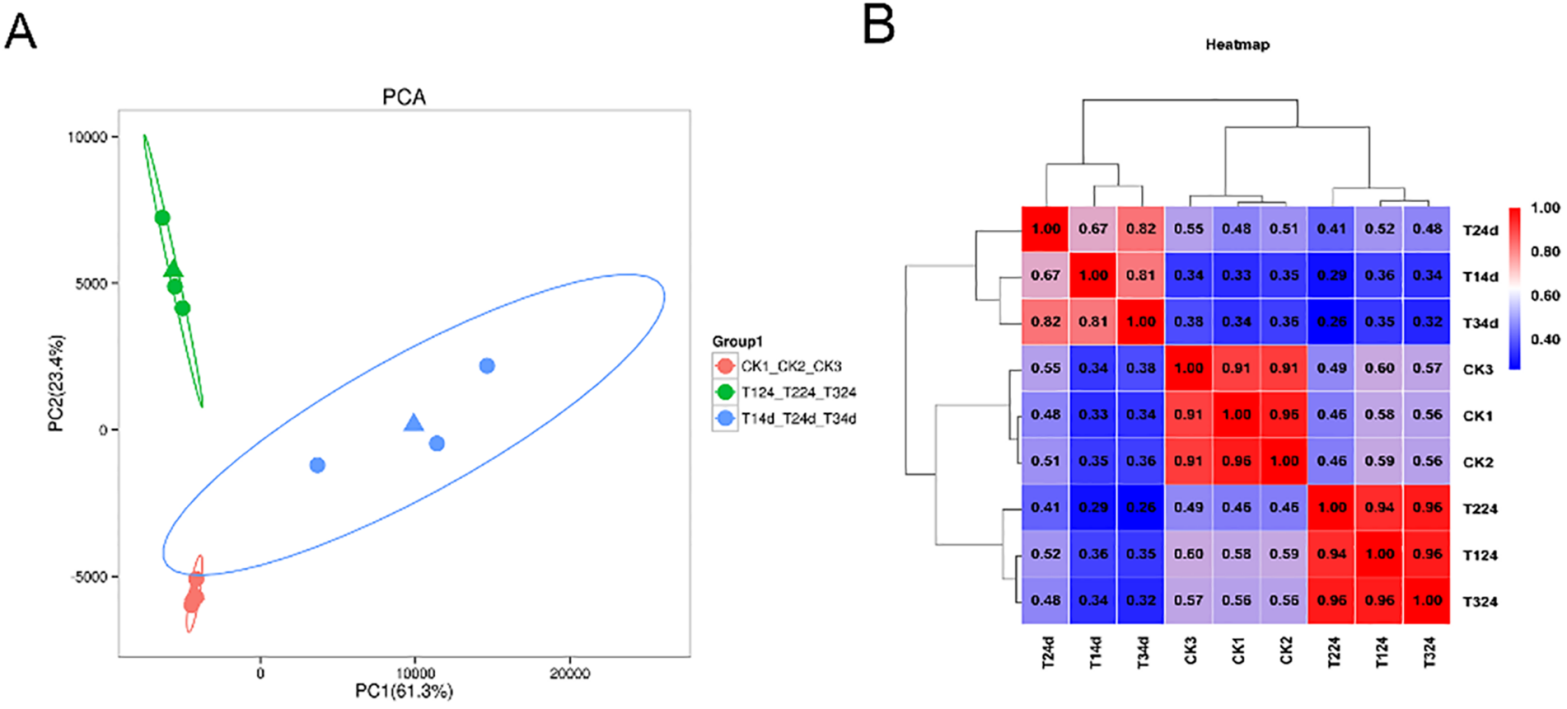
Correlation analysis and principal component distribution. **(A)** PCA score plot: The first two principal components accounted for 61.3% (PC1) and 23.4% (PC2) of total variance, with dashed ellipses indicating 95% confidence intervals. **(B)** Pearson correlation heatmap: Color gradient reflects expression profile similarity between samples (0.34-1.00). Notes: T124/T224/T324: Combined treatment groups at 24 hours post-inoculation by *S. rolfsii*. T14d/T24d/T34d: Combined treatment groups at 4 days post-inoculation by *S. rolfsii*. CK1/CK2/CK3: Biological replicate control groups (n=3).

This study performed original data processing and quality control on nine alfalfa samples, including those inoculated for 24 hours 4 days, and the controls to explore the response mechanism of alfalfa to *S. rolfsii*. Approximately 9.56 GB of raw data were obtained for each sample. The percentage of clean reads from the transcriptome of both uninoculated and inoculated samples exceeded 90%. Using TopHat2 upgrade HISAT2 (http://ccb.jhu.edu/software/hisat2/index.shtml) software, the filtered reads were aligned to the *M. sativa* cv. XinJiangDaYe reference genome. In this reference genome of *M. sativa* cv. XinJiangDaYe, the alignment efficiency ranged from 88.13 to 91.67%, with most sequences mapped to exons (77.10%), followed by introns (19.26%) and only a small portion mapped to intergene regions (3.64%). Principal component analysis (PCA) reveal that replicate samples from the same group were tightly clustered, indicating low variability within biological replicate. The transcriptomic data showed significant differences across time points among different groups, suggesting that infection caused significantly different gene expression (**Figure 4A**). The Spelson correlation coefficients of biological replicates for all samples were approximately >0.8 (**Figure 4B**), indicating a high correlation between sequencing replicates.

### 3.4 DEGs in alfalfa 24 hours and 4 days after pathogen inoculation

**Figure 5:**
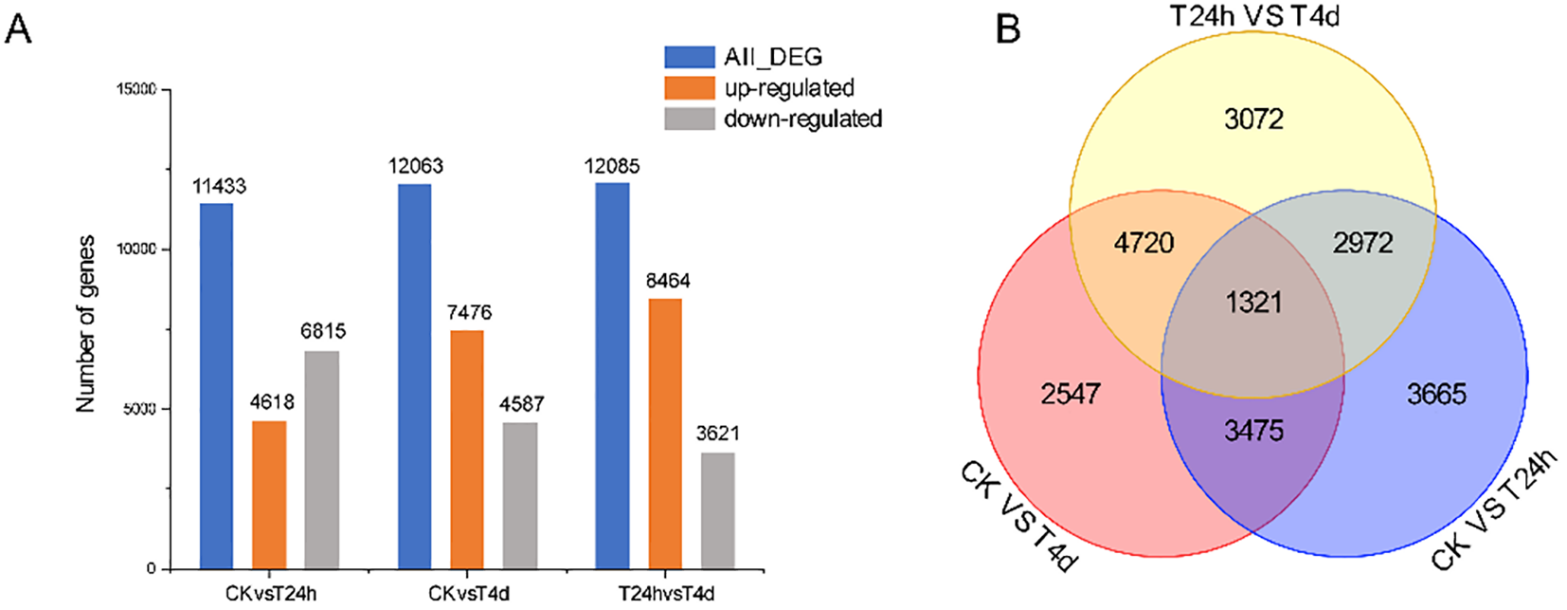
The DEGs between CK vs T24 h, CK vs T4 d, and T24 h vs T4 d in *S. rolfsii* infected alfalfa. **(A)** The DEGs in CK vs T24 h, CK vs T4 d, and T24 h vs T4 d, indicated the up-regulated and downregulated genes. **(B)** The DEGs between CK vs T24 h, CK vs T4 d, and T24 h vs T4 (d) Note: FC≥2 and FDR<0.05 were used as screening criteria.

A pairwise comparison of DEGs between control and inoculated samples were conducted to identify genes involved in alfalfa’s response to *S. rolfsii* infection. The samples were categorized into three comparison groups: CK vs T24 h, CK vs T4 d, and T24 h vs T4 d. In the CK vs. T24 h comparison, 11,433 DEGs were identified, including 4,618 up-regulated and 6,815 down-regulated genes. The CK vs. T4 d group contained 12,063 DEGs, with 7,476 up-regulated and 4,587 down-regulated genes. Lastly, the T24 h vs. T4 d comparison revealed 12,085 DEGs, comprising 8,464 up-regulated and 3,621 down-regulated genes (**Figure 5A**). The number of DEGs increased gradually over the inoculation time. After 4 dpi, the number of DEGs exceeded that at 24 h, and each time point had specific DEGs (**Figure 5B**). The three groups shared 1321 DEGs, indicating that these genes are continuously changing during the alfalfa-*S. rolfsii* interaction, possibly because of the resistance of alfalfa to *S. rolfsii*.

**Supplementary Table 3** shows the top 30 DEGs at 24 hpi and 4 dpi. At 24 hpi, 11 of the first 30 genes were up-regulated, and 19 were down-regulated. Notably, genes associated with disease resistance, such as MS. gene87071 and F-box/LRR-repeat protein, as well as major latex protein genes like MS. gene59838 and MLP-like protein28, were down-regulated. Additionally, defense-related genes, including gibberellin-regulated protein 13 (MS. gene38821) and α-dioxygenase-1 (MS. gene20637), were detected 24 hpi (early inoculation). α-dioxyenase1 detected about functional genes that promote cell growth and cell wall relaxation (Ms. gene055820/Ms. gene043446, expansin-B3. MS. gene55263, and expansin-A15). At 4 dpi, *S. rolfsii* infection differentially expressed only the O-methyltransferase3 gene (MS. gene61087, probably O-methyltransferase3) and the RRM/RBD/RNP motion-family protein of the RNA-binding protein gene (MS. gene045372, probably O-methyltransferase3). Moreover, two RNA-binding RRM/RBD/RNP motif family protein genes were down-regulated at 4 dpi, while the others were up-regulated. *S. rolfsii* infection up-regulated pathogenesis genes such as pathogenesis genes (*Medicago_sativa*_newGene_13595, Newgene_13595, related protein1), peroxidase genes (MS. gene61089, MS. gene006132, peroxidase 12-like protein, and polygalacturonase inhibitor1-like (MS. gene062490, polygalacturonase) were at 4 dpi. A defense-related gene, 2-hydroxyisoflavanone synthase (MS. gene39536, 2-hydroxyisoflavanone synthase), was also up-regulated.

### 3.5 GO and KEGG classification analysis of differentially expressed genes in alfalfa

**Figure 6:**
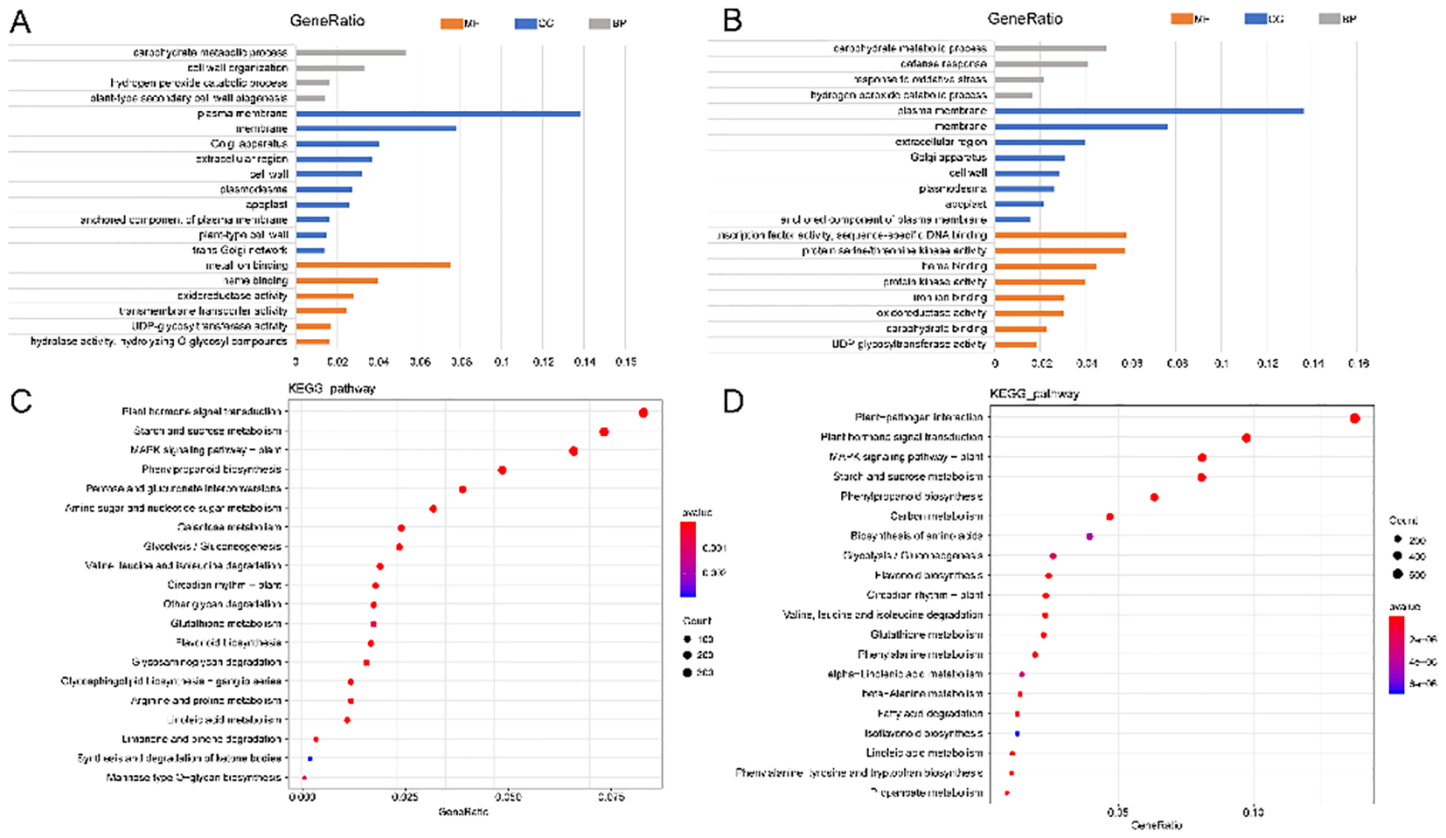
GO and KEGG enrichment analysis of CK vs 24 h and CK vs 4 d. **(A, B)** GO enrichment analysis of CK vs 24 h and CK vs 4 d. Color gradient: GeneRatio values (blue →red=0→0.16). Asterisks: significantly enriched terms (*p*<0.001). **(C, D)** KEGG enrichment analysis of CK vs 24 h and CK vs 4 d. Bubble size: Number of DEGs (diameter ∝ Count). Color intensity: -log10 (*p*value) (blue →red=significance).

Among the top 20 enriched GO terms at 24 hpi, ten belonged to the cellular component category, with DEGs primarily associated with the plasma membrane (GO:0005886) and membrane (GO:0016020). Six GO terms were enriched in the molecular function category, mainly metal ion binding (GO:0046872) and heme binding (GO:0020037). The biological process category included four enriched GO terms, primarily related to carbohydrate metabolism (GO:0005975) and cell wall organization (GO:0071555) (**Figure 6A**).

At 4 dpi, the top 20 enriched GO terms included eight for cell component categories, mainly plasma membrane (GO: 0005886) and membrane (GO: 0016020). Eight GO terms enriched the molecular function category, mainly transcription factor activity, sequence-specific DNA binding (GO: 0003700), and protein serine/threonine kinase activity (GO: 0004674). Four GO items enriched the biological process category, mainly the carbohydrate metabolism process (GO: 0005975) and defense response (GO: 0006952) (**Figure 6B**).

KEGG enrichment analysis revealed that most of these genes enriched plant-pathogen interaction (KO04626), phytohormone signal transduction (KO04075), starch and sucrose metabolism (KO00500), MAPK signaling pathway-plant (KO04016), phenylpropanol biosynthesis (KO00940), and pentose and grape uronic acid interconversion (KO00040) (**Figures 6C, D**). These results indicate that these genes were in a state of continuous change during the interaction between alfalfa and *S. rolfsii*, potentially contributing to alfalfa’s resistance mechanisms against the pathogen.

### 3.6 Key modules in the weighted gene co-expression network at 24 h and 4 d after *Sclerotium rolfsii* infection of alfalfa

A weighted correlation network constructed using the DEGs from nine samples, generating 10011 genes. The objective was to identify the resistance genes of alfalfa against the pathogen *S. rolfsii*, Hclust function in R, and the average method was used to cluster the samples and detect outliers (**Supplementary Figure 1A**). Some samples were obviously divided into two clusters. **Supplementary Figure 1C** shows the TTe heat map of the magenta module and the bar chart of the module characteristic gene expression value. The expression of TTe in the three repetitive module characteristic genes treated by T4 d was higher than in the other treatments. Power 1 was used to calculate the adjacency, and the adjacency was converted into TOM (Topological overlap matrix) to obtain the hierarchical clustering tree (tree graph) of genes that identify the nine modules. Additionally, modules with very similar expression profiles (at a 0.25 cut-off height) were merged, displaying the gene distribution of each sample in each module in the heat map (**Supplementary Figure 1D**). Finally, WGCNA generated three modules, two having opposite expression patterns at different times. The magenta module had the most WGCNA-enriched genes (4039) (**Supplementary Figure 1B**).

**Figure 7:**
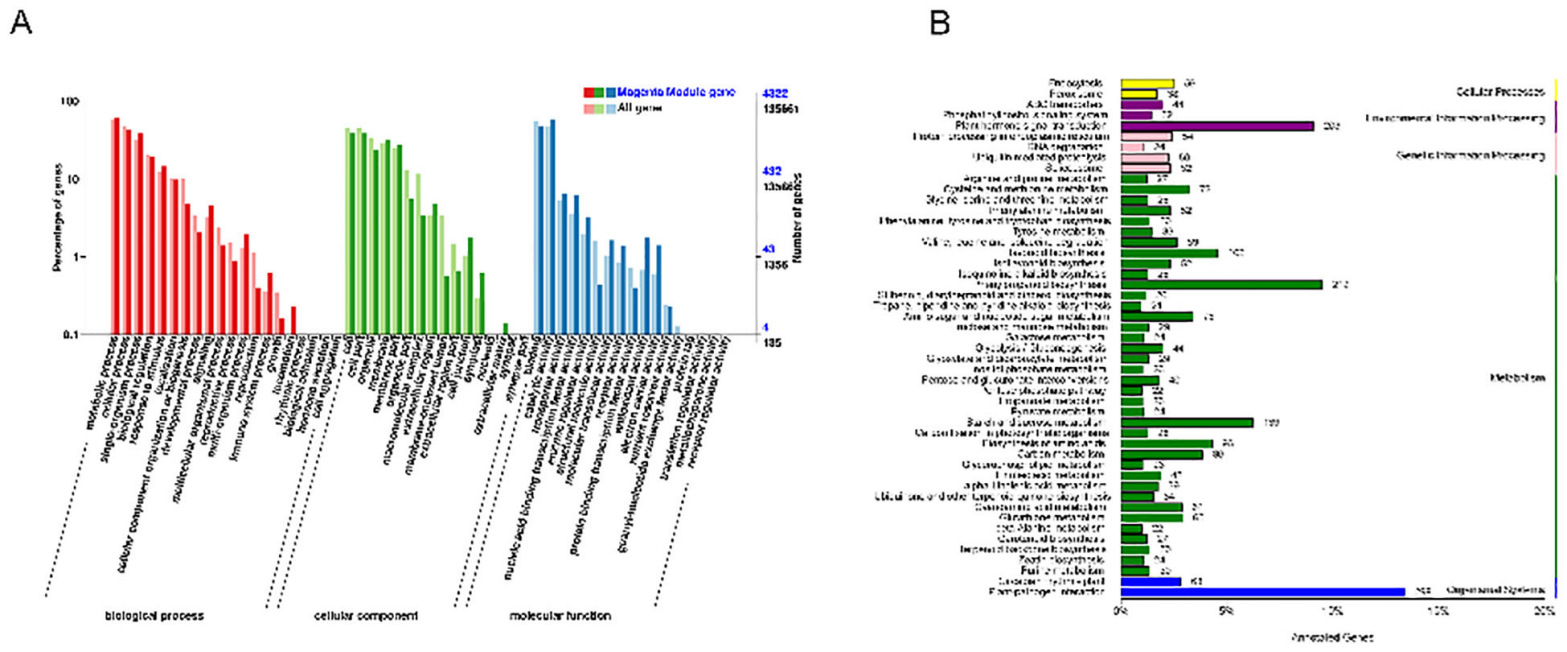
Analysis of highly correlated T4 d GO and KEGG channels of the magenta module. **(A)** Statistical plot of GO second-level node annotation for differentially expressed genes. The x-axis represents GO categories, the left y-axis shows the percentage of gene counts, and the right y-axis shows the gene count. **(B)** DMR gene number in specified annotation KEGG pathways are classified according to the pathway types in KEGG. The y-axis represents the names of KEGG metabolic pathways, and the x-axis shows the number of genes annotated to each pathway and their proportion relative to the total number of annotated genes.

The GO and KEGG pathways enriched by T4 d, which is highly correlated with the magenta module, explained the resistance response mechanism. TTe results of the top 20 GO showed that genes in the magenta module enriched GO:0008150 (biological process), GO: 0008152 (metabolic process), GO: 0009987 (cellular process), GO: 0044699 (single-organism process), and GO: 0071704 (organic substance metabolic process) (**Figure 7A**). Importantly, KEGG pathway analysis showed that most of the DMR (Differentially Methylated Region) genes in the magenta module enriched the first four pathways of plant-pathogen interaction (KO04626) (**Figure 7B**), MAPK signaling pathway-plant (KO04016), phenylpropanoid biosynthesis (KO00940), plant hormone signaling (KO04075) (**Supplementary Table 4**).

**Figure 8:**
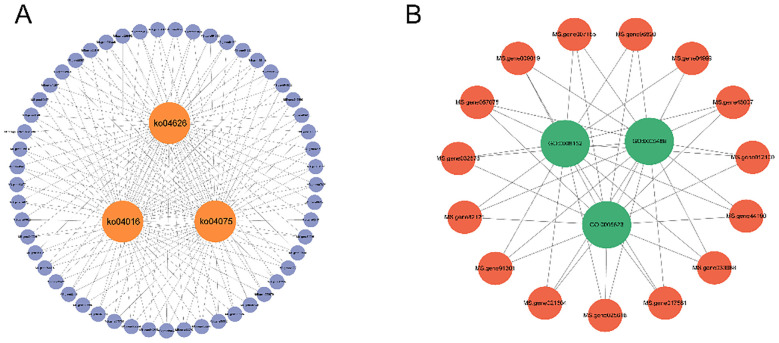
Key GO terms and central genes in KEGG pathways in the 4-day time module of *S. rolfsii* infection in alfalfa under the magenta condition. **(A)** The three KEGG pathways and their associated hub genes. **(B)**The three GO enrichments and their associated hub genes.

An in-depth analysis of the significant three enriched GO terms and KEGG pathways identified the critical genes, specifically, the shared gene sets among the top-ranked GO terms and KEGG pathways. The resulting gene sets were visualized using Cytoscape 3.91, with the KEGG-based network (**Figure 8** and GO-based network (**Figure 8B**). The majority of genes intersecting KEGG pathways plant-pathogen interaction (KO04626), plant hormone signaling (KO04075), and MAPK signaling pathway−plant (KO04016) belong to the PR-1(MS. gene038936, MS. gene002295, and MS. gene00249) family. Among the genes intersecting GO terms, metabolic process (GO: 0008152), cell (GO: 0005623), binding (GO: 0005488), PPR (MS. gene96820, MS. gene009019, MS. gene43007, MS. gene007165, MS. gene82121, and MS. gene025618), protein family and f-box protein (MS. gene033988, MS. gene012160, MS. gene032676, MS. gene44190, and MS. gene017581) were the most enriched, followed by Zinc finger protein (MS. gene04999, MS. gene91301, and MS. gene021604).

### 3.7 Four important KEGG pathways collectively respond to *S. rolfsii* infection

**Figure 9:**
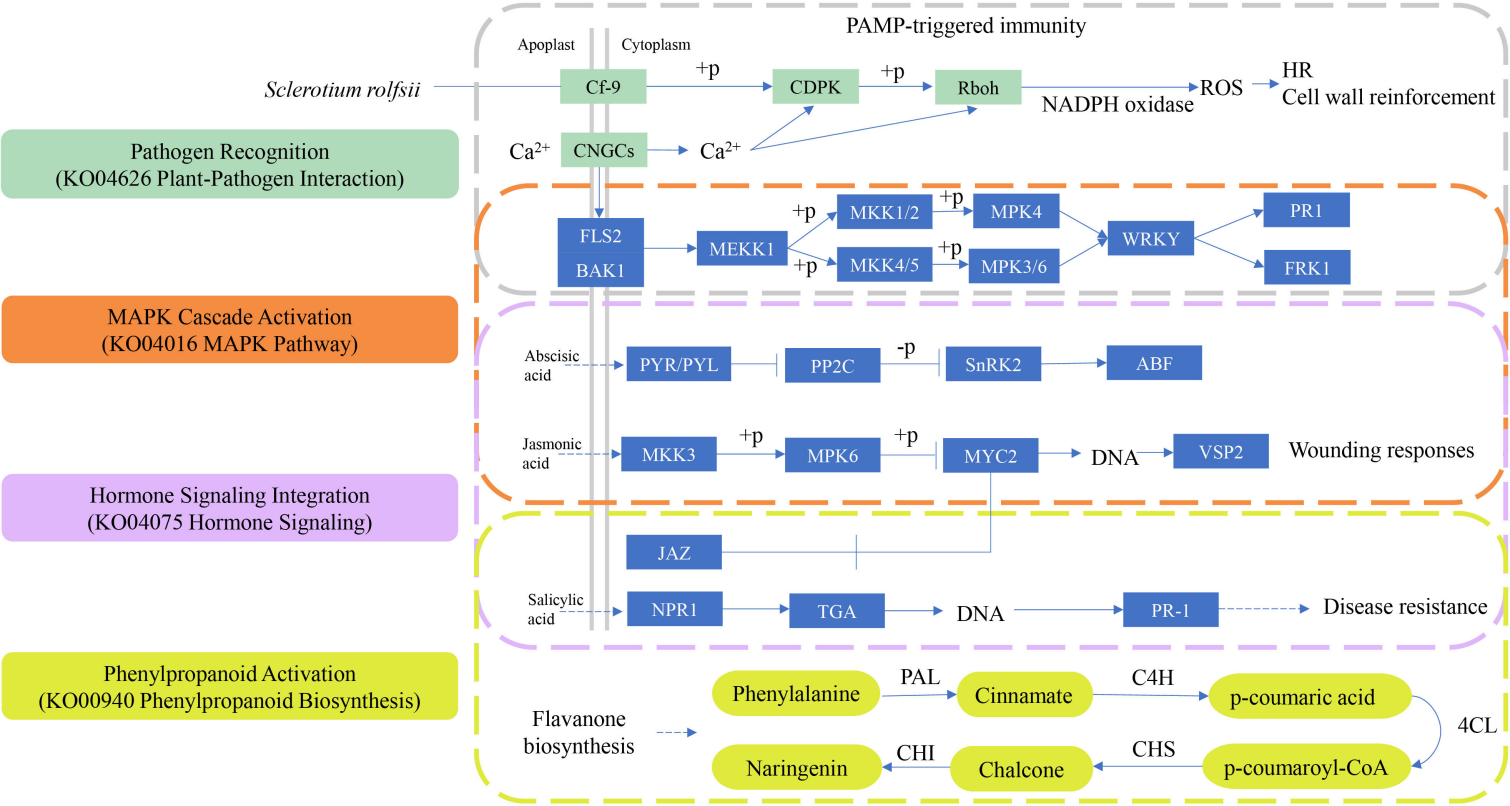
The four key KEGG pathways in plant disease resistance. From top to bottom, the pathways are: Plant-pathogen interaction (KO04626), MAPK signaling pathway- plant (KO04016), Plant hormone signaling (KO04075), Phenylpropanoid biosynthesis (KO00940). PAL, phenylalanine ammonia-lyase; C4H, cinnamate-4-hydroxylase; 4CL, 4-coumarate-CoA ligase; CHS, Chalcone Synthase; CHI, Chalcone Isomerase.

During *S. rolfsii* infection, plants orchestrate a sophisticated defense network integrating multiple signaling pathways (**Figure 9**). Initial pathogen recognition through membrane-localized pattern recognition receptors (PRRs) in the plant-pathogen interaction pathway (KO04626) (**Supplementary Figure 2**) triggers rapid calcium influx and reactive oxygen species (ROS) burst. This activates a phosphorylation cascade in the MAPK signaling pathway (KO04016) (**Supplementary Figure 3**), where sequential activation of MEKK1-MKK4/MKK5-MPK3/MPK6 modules occurs within 15-30 minutes post-infection. Phosphorylated MPK3/MPK6 directly regulates WRKY transcription factors, inducing expression of phenylpropanoid biosynthesis genes (KO00940) (**Supplementary Figure 5**) such as PAL (phenylalanine ammonia-lyase) and 4CL (4-coumarate-CoA ligase). Concurrently, the plant hormone signaling pathway (KO04075) (**Supplementary Figure 4**) coordinates defense prioritization through SA-JA-ET crosstalk: MPK4-mediated degradation of JAZ repressors enhances JA-responsive PDF1.2 expression, while NPR1 oligomerization facilitates SA-dependent PR gene activation. This multipathway synergy results in lignin deposition and phytoalexin accumulation, effectively containing fungal spread. Negative feedback occurs through MAPK phosphatase induction and JA signaling attenuation via JAZ1 restabilization, preventing excessive resource allocation to defense responses.

### 3.8 Transcription factors involved in the defense response after S. rolfsii infection

**Figure 10:**
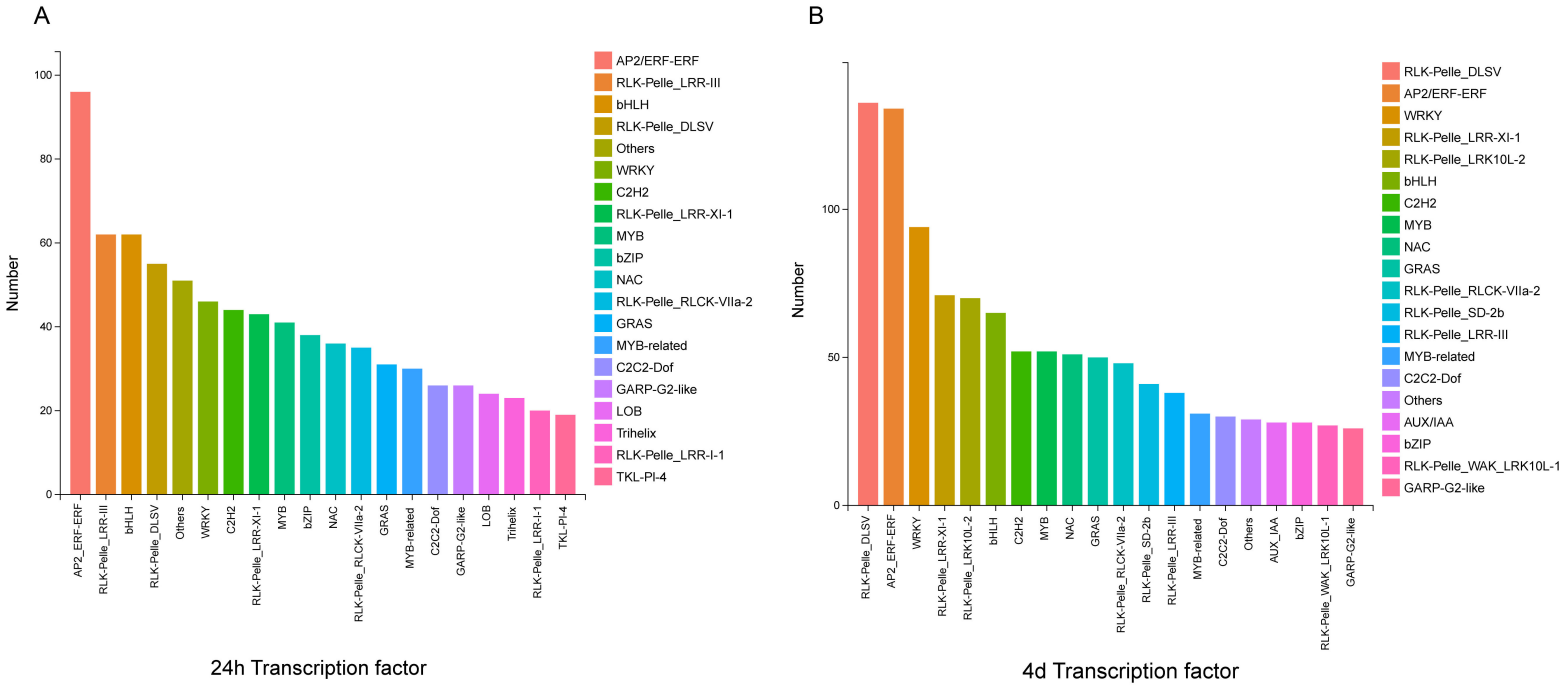
The major families of TFs of DEGs at 24 hours and 4 days after *S. rolfsii* infection. The left image shows the major families of TFs of DEGs 24 hours after *S. rolfsii* infection. The right image shows the major families of TFs of DEGs 4 days after *S. rolfsii* infection.

Transcription factors may positively or negatively regulate downstream defense genes (Javed et al., 2020; Javed and Gao, 2023). In various signaling pathways, TFs regulate and function at different stages of the complex assembly (Baillo et al., 2019). In this experiment, the top 20 most enriched transcription factor families were selected by predicting TFs. The DEGs at 24 h and 4 d were mainly divided into the following transcription factor families: RLK-Pelle_ LRR-II, bHLH, RLK-Pelle_ LRR-III, RLK-Pelle_ DLSV, and WRKY (**Figure 10**). The number of TFs in AP2_ERF-ERF, RLK-Pelle_ DLSV. LRR-III, WRKY, and bHLH families had the highest number of TFs. Among these TFs, although WRKY TFs are not the most prominent, notably, the WRKY TFs family is involved in regulating four important metabolic pathways: plant-pathogen interaction (KO04626), MAPK signaling pathway-plant (KO04016), phenylpropanoid biosynthesis (KO00940), and plant hormone signaling (KO04075).

### 3.9 Real-time fluorescence quantitative PCR analysis

**Figure 11:**
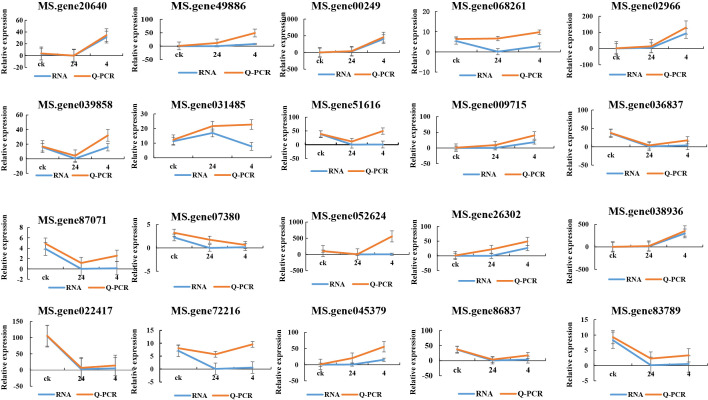
Twenty genes that are potentially associated with plant-pathogen interactions were selected for qPCR validation to verify the accuracy of transcription data. X-axis: represents the experimental groups, which are the CK (control group), 24 hours post-infection (24 hpi), and 4 days post-infection (4 dpi). Y-axis: “Relative expression” indicates normalized expression levels.

Twenty genes, including pathogenesis-related protein 1 (MS. gene00249), O-methyltransferase (MS. gene26302), and chalcone synthase 2 (MS. gene49886) that are potentially associated with plant-pathogen interactions were selected for qPCR validation to verify the accuracy of transcription data. The results showed consistent expression levels with the FPKM values from RNA-seq, thereby confirming the accuracy of the transcriptome analysis (**Figure 11**).

## 4 Discussion

### 4.1 The significance of weighted gene co-expression network analysis and hub gene identification

A WGCNA determined the molecular mechanisms between alfalfa-*S. rolfsii* interaction (El-Sharkawy et al., 2015; Wang et al., 2024a) using the obtained transcriptome data. WGCNA identified three modules, and the magenta module, most enriched with DEGs related to disease resistance, was selected for further analysis. Furthermore, correlation analysis identified GO and KEGG pathways associated with the alfalfa-pathogen interaction. Three GO terms and three KEGG pathways were selected as central hubs, identifying several key protein families, including PR-1, PPR, and F-box. These genes are potentially pivotal for elucidating the mechanisms underlying disease resistance. The Pathogenesis-Related Protein 1 (PR-1) family is a crucial class of proteins plants produce in response to pathogen invasion. Thus, the expression of PR-1 genes is a marker in studies of plant salicylic acid-mediated disease resistance (Breen et al., 2017).

Although the exact molecular mechanisms of PR-1 remain unclear, it may inhibit pathogen growth or activity directly. Additionally, PR-1 shares structural similarities with steroid-binding proteins, indicating a potential role in signal transduction during pathogen attack (van Loon et al., 2006). The specific role of PPR proteins in alfalfa disease resistance remains unclear. In other plants, PPR proteins play critical roles in disease resistance. For instance, in *Arabidopsis thaliana*, the mitochondria-localized PPR (Yang et al., 2022b) protein RTP7 regulates the activity of mitochondrial complex I, affecting the production of mitochondrial reactive oxygen species (mROS), thereby mediating broad-spectrum resistance to multiple pathogens. This function deepens our understanding of these metabolic pathways, providing a solid theoretical foundation for future research on their roles.

### 4.2 The plant hormone pathways are associated with the susceptibility of alfalfa to pathogens

Plant hormones play different roles in plant infection by pathogenic bacteria (Pieterse et al., 2012; Chanclud and Morel, 2016). When invaded by pathogenic fungi, plants stimulate the expression of resistance genes through signaling molecules such as jasmonic acid (JA) (Wang et al., 2022), salicylic acid (SA), and ethylene (ETH) and complex signaling networks to develop disease resistance to pathogenic fungi (Di et al., 2017). This study identified 439 and 472 DEGs related to plant hormone signaling pathways at 24 hpi and 4 dpi, respectively. The DEGs enrich auxin, abscisic acid (ABA), gibberellin, salicylic acid, jasmonic acid, and ethylene pathways (Aparicio and Pallás, 2017). ABA (Raghavendra et al., 2010) prevents pathogen entry by closing stomata (Melotto et al., 2024) or antagonizing SA/JA to promote plant susceptibility. In response to environmental stress, plants release JA (Ruan et al., 2019) signaling molecules faster, suggesting their crucial role in plant disease resistance. Moreover, SA acts as an *NPR1* precursor and transcriptional activator. *NPR1* controls SA-mediated (Ding et al., 2018) gene expression and disease resistance. After infecting the susceptible wheat variety, Roblin, *Fusarium graminis*, accumulated a large amount of Indole acetic acid (IAA) (Qi et al., 2016). SA is a pivotal hormone in plant defense, particularly in resistance to biotrophic pathogens. Besides, SA triggers systemic acquired resistance (SAR) by activating downstream TFs, such as *NPR1*, promoting PR-1 gene expression. Elevated expression of PR-1, a molecular marker for SA-mediated immunity, often correlates with enhanced disease resistance in plants (Durrant and Dong, 2004). The transcriptome study showed that *S. rolfsii* up-regulated IAA synthesis gene 24 h after infecting alfalfa. Therefore, the IAA pathway is associated with alfalfa susceptibility.

Increased auxin levels during pathogen infection stimulate cell wall loosening, change the membrane permeability, and promote stomatal opening, facilitating pathogen transmission. Elevated auxin levels also induced the expression of numerous auxin-responsive genes, including members of the Aux/IAA and GH3 gene families (Chen et al., 2007). In this study, *S. rolfsii* up-regulated the expression of *GH3*, possibly because of IAA accumulation (Xie et al., 2022). Thus, IAA accumulation increased *S. rolfsii* pathogenicity. In addition, the JasmonateZIM-domain (JAZ) protein is a suppressor of the jasmonate signaling pathway, and downregulating JAZ-related genes increases JA content (Pauwels and Goossens, 2011). JA and SA are antagonistic; thus, increasing the JA content inhibits SA synthesis and signal transduction (Thaler et al., 2012; Yuan et al., 2017). SA is among the key signaling molecules that activate the defense response of plants against pathogens (Dempsey and Klessig, 2017). ABA is also primarily associated with responses to abiotic stress and can indirectly influence PR-1 expression by modulating the SA signaling pathway. Inhibiting SA signal transduction reduces the immunity of plants to *S. rolfsii*. Therefore, *S. rolfsii* showed more virulence. Further analysis of key genes associated with plant hormone biosynthesis and signal transduction is essential for elucidating their specific roles of plant hormones in the alfalfa-pathogen interaction.

### 4.3 The phenylpropanoid biosynthesis pathway plays a pivotal role in plant defense mechanisms against pathogens

The phenylpropanoid biosynthesis pathway (KO00940) functions as a central defense mechanism in plant immunity, enzymatically converting L-phenylalanine into specialized metabolites with antimicrobial properties. Within this biochemical cascade, lignin deposition establishes physical barriers against pathogen penetration, while flavonoid derivatives demonstrate dose-dependent antimicrobial activity through reactive oxygen species modulation and virulence factor inhibition (Dixon et al., 2002). When *S. rolfsii* infects alfalfa, cells surrounding infection sites accelerate lignin synthesis, leading to increased lignification. This structural modification effectively impedes fungal hyphal penetration or enzymatic degradation. Pathogen-associated molecular patterns (PAMPs) trigger immune signals that upregulate key lignin biosynthesis enzymes (phenylalanine ammonia-lyase, PAL) via the JA pathway. Lignin deposition involves peroxidase-catalyzed oxidation reactions that generate ROS, which promote lignin polymerization and directly damage pathogens (Yadav et al., 2020). Flavonoids play a crucial role in the plant’s immune defense. As secondary metabolites, flavonoids possess various bioactivities, including antioxidant, antimicrobial, and antifungal properties, which help plants resist pathogen attacks. Some flavonoids, such as quercetin and kaempferol, exhibit direct antifungal effects. They can inhibit fungal growth or disrupt fungal cell structures by interacting with the fungal cell membrane, preventing the spread and establishment of the pathogen. Flavonoids can trigger the expression of defense-related genes in plants, activating immune responses (Vogt, 2010). Flavonoids influence the levels of plant hormones, particularly auxins, abscisic acid (ABA), and salicylic acid (SA). In the phenylpropanoid biosynthesis pathway, enzymes such as PAL, C4H, 4CL, CHS, and CHI play crucial roles. Further analysis of the genes encoding these enzymes is essential for a deeper understanding of the disease resistance mechanisms in alfalfa.

### 4.4 WRKY TFs integrate multiple signaling pathways to coordinate plant defense responses against various stresses

WRKY transcription factors (TFs) are one of the largest transcription factor families in plants and play a crucial role in plant defense against both biotic and abiotic stresses (Wang et al., 2024b). They interact with various signaling pathways, including MAPK cascades, to enhance the plant’s immune response. Studies have shown that MAPK-phosphorylated WRKY TFs can regulate NADPH oxidase in Nicotiana benthamiana, thereby controlling the downstream burst of ROS (Adachi et al., 2015). WRKY TFs are involved in regulating plant hormone signaling. For example, in Arabidopsis, WRKY28 directly binds to the promoter of the IC synthase 1 (ICS1) gene, which activates the expression of ICS1, an important step in SA biosynthesis (van Verk et al., 2011). Additionally, WRKY TFs are involved in the phenylpropanoid biosynthesis pathway, which is essential for plant development and survival. This pathway contributes to the production of metabolites that play important roles in the defense against pathogen infection (Javed and Gao, 2023). Although WRKY TFs have been extensively studied in relation to plant defense, there are still many aspects that warrant further investigation. For example, the expression of WRKY TFs and their mechanisms of stress resistance under the combined effects of biotic and abiotic stresses.

## 5 Conclusions

This study investigated the mechanism of plant-pathogen interaction during the early stages of *S. rolfsii* infection in alfalfa using transcriptome sequencing at different infection time points. The number of DEGs increased with the duration of inoculation. Specifically, they were identified in CK vs. T24 h as having 11,433 DEGs and CK vs. T4 d as having 12,063 DEGs. Most DEGs primarily enriched phenylpropanoid biosynthesis, the MAPK signaling pathway in plants, plant-pathogen interactions, carbon metabolism, and the plant hormone signaling pathway. The plant-pathogen interaction and plant hormone signaling pathways are key players in the disease-resistance response mechanism of alfalfa. Furthermore, three protein families, PR-1, F-box, and the Zinc finger, are potentially critical to the disease resistance mechanism of plants. In the phenylpropanoid biosynthesis pathway, genes such as *PAL*, *C4H*, *4CL*, *CHS*, and *CHI* play crucial roles. Further analysis of these genes is essential for a deeper understanding of plant-pathogen interaction mechanisms. Numerous TFs from the WRKY, AP2/ERF-ERF, and bHLH families may be involved in plant-pathogen interactions. Among plant regulatory components, the WRKY TFs family warrants particular attention, functioning as a master regulator coordinating multiple disease resistance-associated metabolic pathways. This study provides a new way to study the transcriptomes of alfalfa at different times after inoculation. Further research is needed to reveal the molecular mechanism of DEGs related to the physiological indexes of alfalfa, providing an important theoretical basis for the molecular mechanism of alfalfa disease resistance.

## Data Availability

The datasets presented in this study can be found in online repositories. The names of the repository/repositories and accession number(s) can be found in the article/**Supplementary Material.**
