# Supplementary figures and images for: Transcriptome analysis reveals defense responses of alfalfa seedling roots to Sclerotium rolfsii
Source: Front Plant Sci. 2025 Apr 15;16:1561723. doi: 10.3389/fpls.2025.1561723 (PMC12038447; doi:10.3389/fpls.2025.1561723)

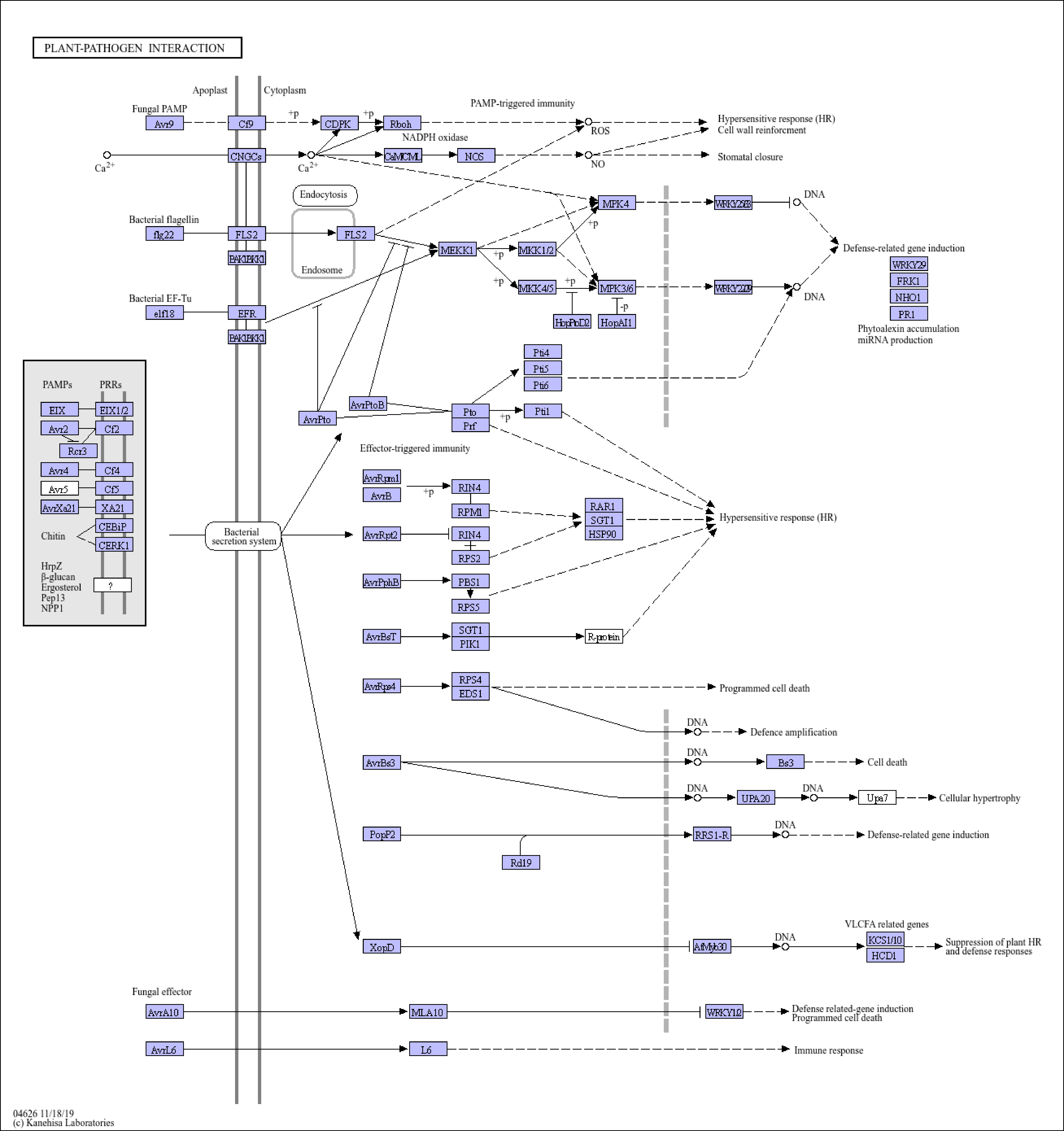

Supplement: Supplementary file 1 [file Image1.jpeg]

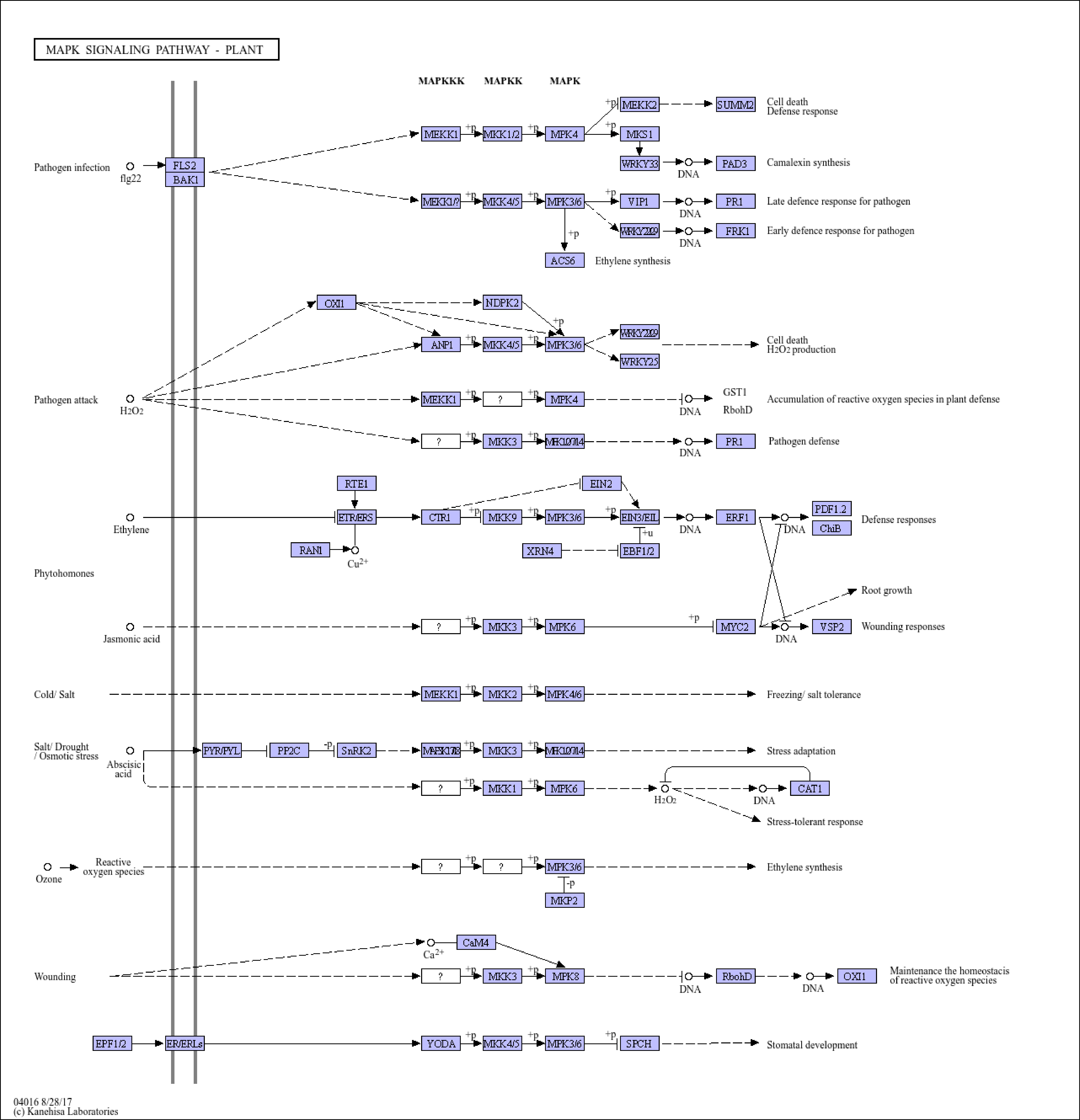

Supplement: Supplementary file 2 [file Image2.jpeg]

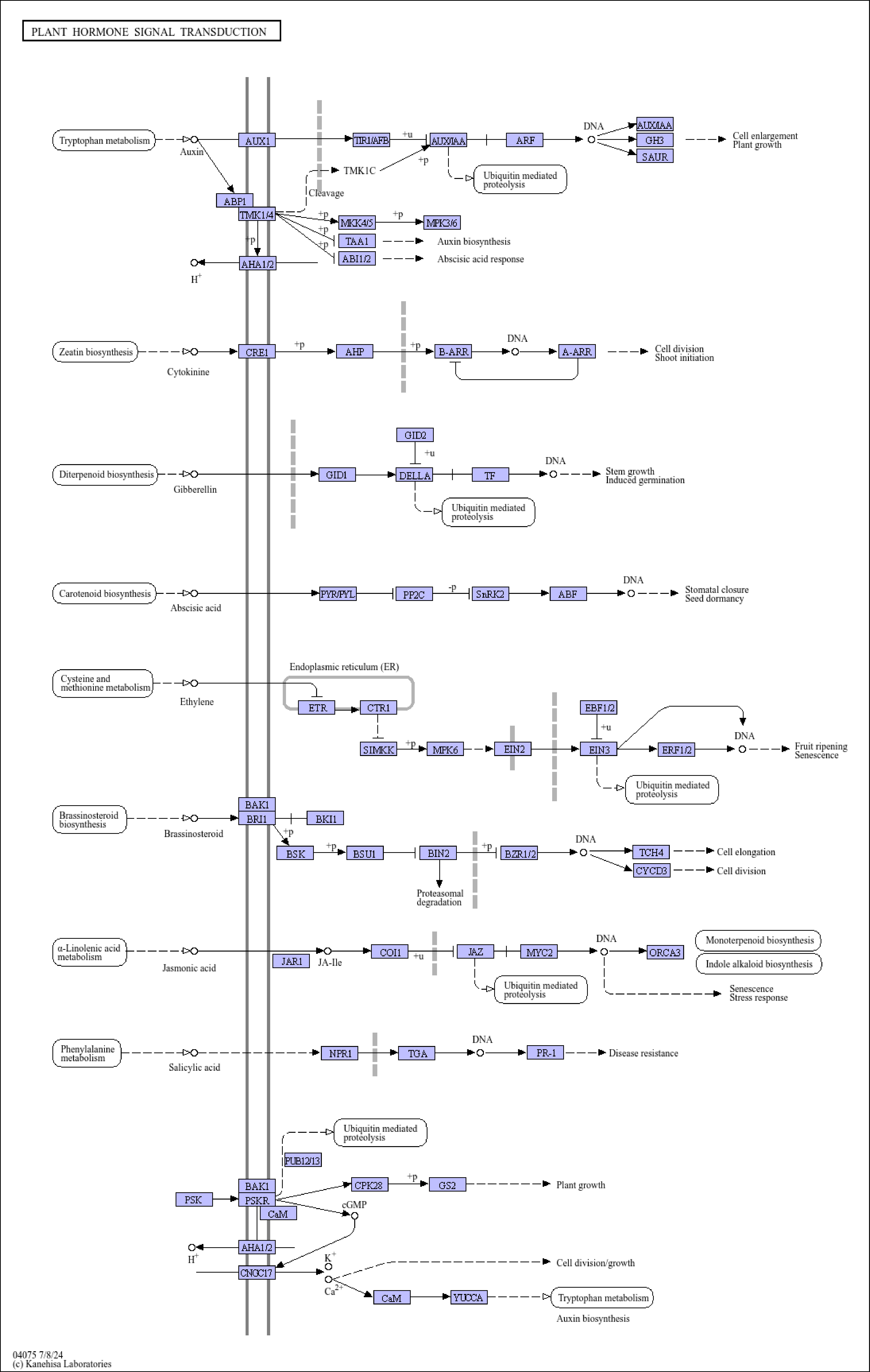

Supplement: Supplementary file 3 [file Image3.jpeg]

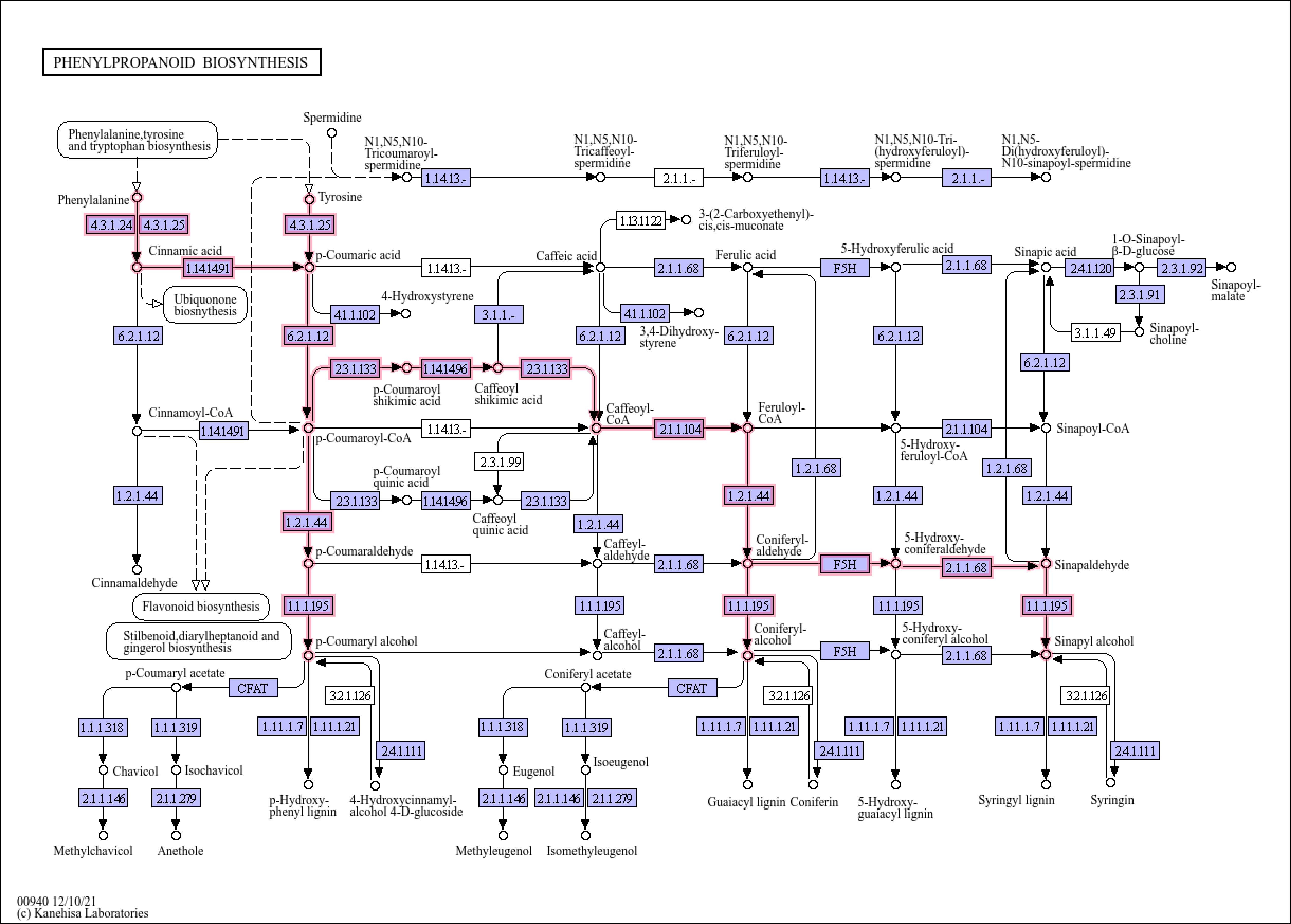

Supplement: Supplementary file 4 [file Image4.jpeg]
