# Supplementary material for: Transcriptome analysis reveals defense responses of alfalfa seedling roots to Sclerotium rolfsii
Source: Front Plant Sci. 2025 Apr 15;16:1561723. doi: 10.3389/fpls.2025.1561723 (PMC12038447; doi:10.3389/fpls.2025.1561723)
Supplement: Supplementary file 5 [file Table1.docx]

**Supplementary Figure 1** Key modules in the weighted gene co-expression network (WGCNA) at 24 h and 4 d after Sclerotium rolfsii infection of alfalfa


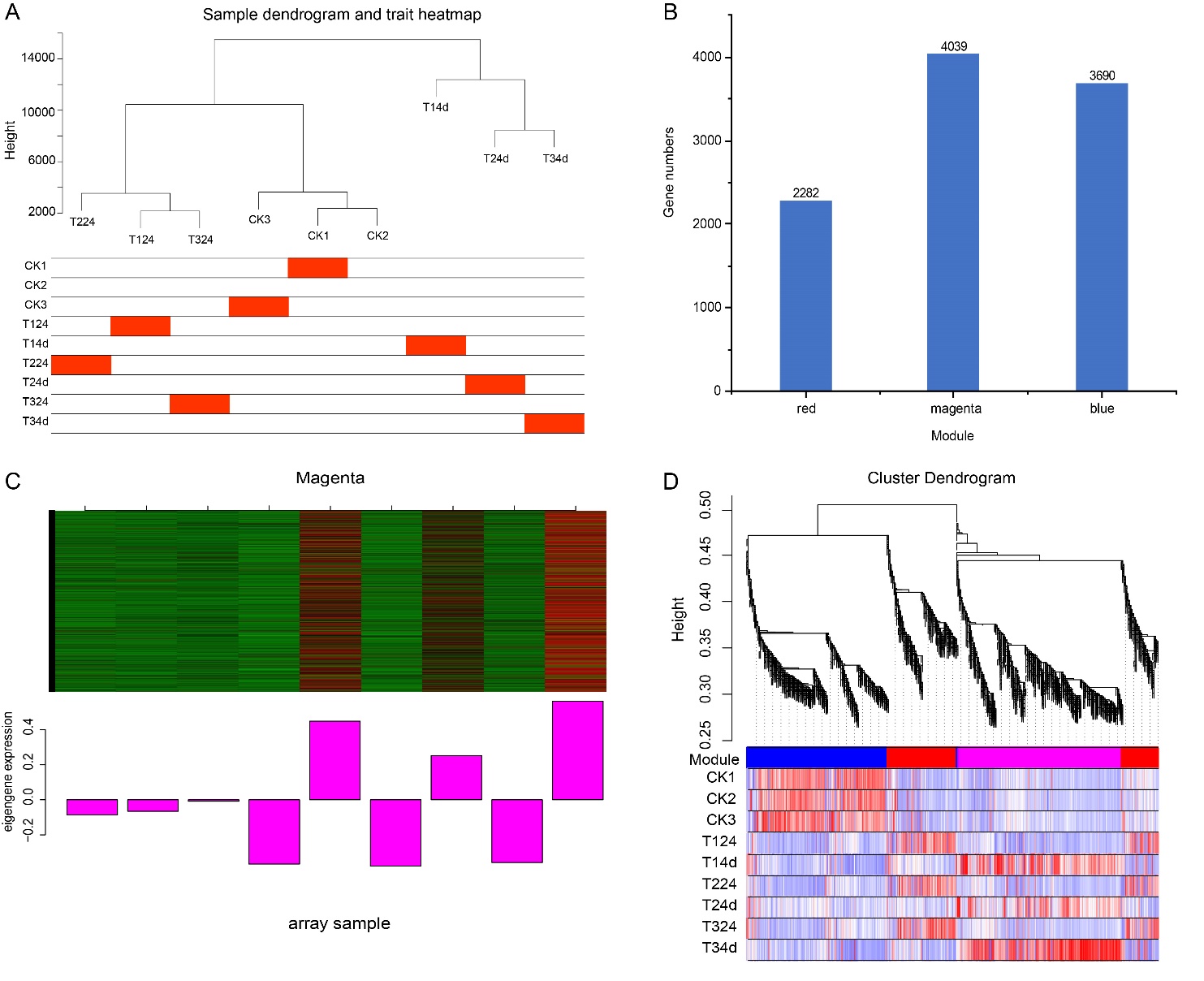


**Figure S1.** **(A****)** Cluster analysis of samples to detect outliers. All samples are located in the clusters and are divided into two clusters. **(B)** The number of genes in each module. **(C)** The heat map and bar chart of gene expression values of module characteristics. **(D)** Gene clustering tree and heatmap of distribution of gene expression between each module and each sample.
