# Supplementary material for: Transcriptome analysis reveals defense responses of alfalfa seedling roots to Sclerotium rolfsii
Source: Front Plant Sci. 2025 Apr 15;16:1561723. doi: 10.3389/fpls.2025.1561723 (PMC12038447; doi:10.3389/fpls.2025.1561723)
Supplement: Supplementary file 6 [file Table2.docx]

Supplementary Material

# Supplementary Tables

**Supplementary Table 2**

Table 1 qRT-PCR primer sequences

| Gene ID | Forward primer sequences (5‘to 3’) | Reverse primer sequences (5‘to 3’) |
| --- | --- | --- |
| *MS-ACTIN* | CTCTCAAGTACCCCATTGAGC | TATTGGCCTTTGGGTTAAGTG |
| MS. gene51616 | GGCGTGTAATAACCTTGATAC | AGAGACCTCCAGAGATATGA |
| MS. gene72216 | GGTCTCCGTAACTCATCA | ATCTTCCACTGCTTCCAA |
| MS. gene022417 | TCATAGCGACTCTTATACTCAA | CTACACCTTGGCATCCTT |
| MS. gene07380 | CTCATCGTGGTTCCTCAA | CGCTCCATCACAGTATCT |
| MS. gene052624 | TCATAGCGACTCTTATACTCAA | CTACACCTTGGCATCCTT |
| MS. gene20640 | TTGACAAGATGGCAATATGG | AGCAGTTCTATATGGATGAGT |
| MS. gene87071 | GCCTTAATCTCACTTCATTGG | GTCAACCGCATAATCTCAAG |
| MS. gene86837 | TCTGCTGTAGTTGCTCAT | ACCATTGTTCTTCCTTCTTG |
| MS. gene036837 | TCTGCTGTAGTTGCTCAT | ACCATTGTTCTTCCTTCTTG |
| MS. gene068261 | GTTCTTGATTCTTCCACACT | GACTTGTTCGGCTGTTAG |
| MS. gene009715 | ACCTGATATTATACACAACTATGG | ATAGATTCATCGGTTAGCATATAC |
| MS. gene039858 | TAACTGAAGAACAAGAGATAACC | ATTAACATAACCATTCCACGAT |
| MS. gene83789 | ATTGTTGCTCATAACTTGATACT | GTCACTAAGGTCACTCCAA |
| MS. gene26302 | AGCCGTACTACATCCAATT | ACTTCTCAATCACCACACT |
| MS. gene038936 | TCACATCTCCTTCCACTC | TGAATCTTGAGGTAGTCTTGT |
| MS. gene00249 | TCACATCTCCTTCCACTC | TGAATCTTGAGGTAGTCTTGT |
| MS. gene49886 | ATGGTGGTGGTAGAGGTA | AGTTTGGTGAGTTGATAATCG |
| MS. gene045379 | GTTGCCAGAAGTTGAGAAG | GAGGAGATGGAATGTCAGT |
| MS. gene02966 | GCCGCTAGGTGTTCATAA | TGACTGTAACTTGTGCTACT |
| MS. gene031485 | GGAGTTGTTCACTTACTTATCTT | GGAGTTGTTCACTTACTTATCTT |

**Supplementary Table 3** The top 30 DEGs in alfalfa at 24 h and 4 d post-inoculation with the pathogen versus the control

| **24 h** | | | | | | |
| --- | --- | --- | --- | --- | --- | --- |
| **Time** |  | **Gene ID** | **NR_annotation** | **Regulated** | **log_2_FC** | **FDR** |
| 24 h |  | MS. gene78072 | 21 kDa protein | up | 7.50 | 1.65E-98 |
| 24 h |  | MS. gene045669 | 21 kDa protein | up | 7.42 | 1.41E-70 |
| 24 h |  | MS. gene38821 | gibberellin-regulated protein 13 | down | -7.44 | 1.76E-41 |
| 24 h |  | MS. gene20640 | alpha-dioxygenase 1 isoform X1 | down | -7.06 | 3.00E-33 |
| 24 h |  | MS. gene20637 | alpha-dioxygenase 1 | down | -6.96 | 2.25E-56 |
| 24 h |  | MS. gene05944 | uncharacterized protein LOC11415745 | up | 6.77 | 4.04E-46 |
| 24 h |  | MS. gene66408 | uncharacterized protein LOC11427632 | up | 6.71 | 1.87E-35 |
| 24 h |  | MS. gene90590 | unknown seed protein USP | down | -6.67 | 4.01E-27 |
| 24 h |  | Medicago_sativa_newGene_26200 | -- | down | -6.66 | 4.93E-28 |
| 24 h |  | MS. gene006767 | thioredoxin-like protein CXXS1 | up | 6.63 | 1.23E-31 |
| 24 h |  | Medicago_sativa_newGene_31574 | uncharacterized protein LOC11427632 | up | 6.53 | 3.03E-62 |
| 24 h |  | MS. gene59838 | MLP-like protein 28 | down | -6.47 | 6.09E-57 |
| 24 h |  | MS. gene062068 | putative guanine deaminase | up | 6.45 | 9.03E-24 |
| 24 h |  | MS. gene001312 | unknown | up | 6.39 | 4.85E-25 |
| 24 h |  | MS. gene055820 | expansin-B3 | down | -6.36 | 6.06E-53 |
| 24 h |  | MS. gene51616 | probable aquaporin NIP-type | down | -6.30 | 3.36E-119 |
| 24 h |  | MS. gene07380 | pectin acetylesterase 10 | down | -6.26 | 3.49E-23 |
| 24 h |  | MS. gene015005 | mitochondrial carrier protein CoAc1 | down | -6.24 | 3.25E-26 |
| 24 h |  | MS. gene37296 | 7-deoxyloganetin glucosyltransferase | up | 6.14 | 1.54E-49 |
| 24 h |  | MS. gene009444 | probable aquaporin NIP-type | down | -6.11 | 1.27E-95 |
| 24 h |  | MS. gene063322 | uncharacterized protein LOC11427632 | up | 6.10 | 5.68E-82 |
| 24 h |  | MS. gene72216 | glyceraldehyde-3-phosphate dehydrogenase A, chloroplastic | down | -5.99 | 3.67E-58 |
| 24 h |  | MS. gene55263 | expansin-A15 | down | -5.97 | 3.80E-38 |
| 24 h |  | MS. gene024334 | glyceraldehyde-3-phosphate dehydrogenase A, chloroplastic | down | -5.92 | 1.80E-53 |
| 24 h |  | MS. gene043446 | expansin-B3 | down | -5.88 | 9.44E-27 |
| 24 h |  | MS. gene87071 | F-box/LRR-repeat protein At3g03360 | down | -5.85 | 1.16E-29 |
| 24 h |  | MS. gene049058 | uncharacterized protein LOC11415745 | up | 5.83 | 1.62E-64 |
| 24 h |  | MS. gene010104 | beta carbonic anhydrase 5, chloroplastic isoform X1 | up | 5.81 | 8.20E-56 |
| 24 h |  | Medicago_sativa_newGene_5933 | uncharacterized protein LOC112421923 | down | -5.80 | 4.19E-31 |
| 24 h |  | MS. gene51913 | FT | down | -5.76 | 8.06E-32 |
| 24 h |  | MS. gene044274 | linoleate 9S-lipoxygenase 1 | down | -5.76 | 2.46E-28 |
| **T4 d** | | | | | | |
| T4 d |  | MS. gene26302 | O-methyltransferase | up | 9.50 | 7.67E-36 |
| T4 d |  | Medicago_sativa_newGene_13595 | pathogenesis-related protein 1 | up | 9.25 | 6.38E-50 |
| T4 d |  | MS. gene009715 | probable O-methyltransferase 3 | up | 9.24 | 2.24E-33 |
| T4 d |  | MS. gene61089 | peroxidase 12-like protein | up | 9.23 | 1.55E-32 |
| T4 d |  | MS. gene006132 | peroxidase 12-like protein | up | 9.20 | 3.02E-32 |
| T4 d |  | MS. gene037463 | probable 2-oxoglutarate-dependent dioxygenase At5g05600 | up | 9.05 | 6.19E-36 |
| T4 d |  | MS. gene038936 | pathogenesis-related protein 1 | up | 8.92 | 3.46E-55 |
| T4 d |  | MS. gene41245 | probable O-methyltransferase 3 | up | 8.79 | 1.75E-32 |
| T4 d |  | MS. gene98524 | abscisic acid and environmental stress-inducible protein | up | 8.70 | 1.04E-112 |
| T4 d |  | MS. gene05141 | probable glutathione S-transferase parA | up | 8.65 | 7.51E-32 |
| T4 d |  | MS. gene062490 | polygalacturonase inhibitor 1-like | up | 8.60 | 1.44E-43 |
| T4 d |  | MS. gene44570 | isoliquiritigenin 2'-O-methyltransferase | up | 8.57 | 1.38E-25 |
| T4 d |  | MS. gene54139 | putative oxoglutarate/iron-dependent dioxygenase, non-heme dioxygenase domain-containing protein | up | 8.40 | 1.76E-24 |
| T4 d |  | MS. gene062068 | putative guanine deaminase | up | 8.31 | 1.15E-26 |
| T4 d |  | MS. gene91233 | O-methyltransferase | up | 8.29 | 5.80E-25 |
| T4 d |  | MS. gene00249 | pathogenesis-related protein 1 | up | 8.22 | 2.62E-110 |
| T4 d |  | MS. gene62755 | -- | up | 8.18 | 4.80E-23 |
| T4 d |  | MS. gene003914 | beta-glucosidase 12 | up | 8.14 | 4.76E-27 |
| T4 d |  | MS. gene016424 | putative major facilitator superfamily | up | 8.02 | 6.62E-22 |
| T4 d |  | MS. gene52695 | dirigent protein 2 | up | 7.96 | 1.32E-21 |
| T4 d |  | MS. gene056022 | probable O-methyltransferase 3 | up | 7.94 | 8.41E-23 |
| T4 d |  | MS. gene030455 | Ty3/gypsy retrotransposon protein | up | 7.93 | 2.84E-21 |
| T4 d |  | MS. gene39536 | 2-hydroxyisoflavanone synthase | up | 7.93 | 3.08E-27 |
| T4 d |  | MS. gene49886 | chalcone synthase 2 | up | 7.83 | 9.90E-29 |
| T4 d |  | MS. gene045372 | RNA-binding (RRM/RBD/RNP motif) family protein | down | -7.78 | 9.88E-25 |
| T4 d |  | MS. gene41247 | O-methyltransferase | up | 7.76 | 4.28E-21 |
| T4 d |  | MS. gene20675 | VQ motif-containing protein 29 | up | 7.75 | 3.65E-21 |
| T4 d |  | MS. gene015272 | VQ motif-containing protein 29 | up | 7.75 | 5.25E-21 |
| T4 d |  | MS. gene052433 | putative SLC26A/SulP transporter | up | 7.73 | 1.27E-34 |
| T4 d |  | MS. gene61087 | probable O-methyltransferase 3 | down | -7.67 | 1.53E-23 |
